# Targeting chondroitin sulfate suppresses macropinocytosis of breast cancer cells by modulating syndecan‐1 expression

**DOI:** 10.1002/1878-0261.13667

**Published:** 2024-05-21

**Authors:** Hung‐Rong Yen, Wen‐Chieh Liao, Chia‐Hua Chen, Ying‐Ai Su, Ying‐Wei Huang, Chi Hsiao, Yu‐Lun Chou, Yin‐Hung Chu, Pin‐Keng Shih, Chiung‐Hui Liu

**Affiliations:** ^1^ Department of Chinese Medicine China Medical University Hospital Taichung Taiwan; ^2^ Chinese Medicine Research Center, and School of Chinese Medicine, College of Chinese Medicine China Medical University Taichung Taiwan; ^3^ Doctoral Program in Tissue Engineering and Regenerative Medicine, College of Medicine National Chung Hsing University Taichung Taiwan; ^4^ Department of Post‐Baccalaureate Medicine, College of Medicine National Chung Hsing University Taichung Taiwan; ^5^ Molecular Medicine Research Center Chang Gung University Taoyuan Taiwan; ^6^ College of Medicine Chung Shan Medical University Taichung Taiwan; ^7^ Department of Surgery China Medical University Hospital Taichung Taiwan; ^8^ School of Medicine China Medical University Taichung Taiwan

**Keywords:** breast cancer, chondroitin sulfate, CHPF, macropinocytosis, syndecan 1

## Abstract

Accumulation of abnormal chondroitin sulfate (CS) chains in breast cancer tissue is correlated with poor prognosis. However, the biological functions of these CS chains in cancer progression remain largely unknown, impeding the development of targeted treatment focused on CS. Previous studies identified chondroitin polymerizing factor (CHPF; also known as chondroitin sulfate synthase 2) is the critical enzyme regulating CS accumulation in breast cancer tissue. We then assessed the association between CHPF‐associated proteoglycans (PGs) and signaling pathways in breast cancer datasets. The regulation between CHPF and syndecan 1 (SDC1) was examined at both the protein and RNA levels. Confocal microscopy and image flow cytometry were employed to quantify macropinocytosis. The effects of the 6‐*O*‐sulfated CS‐binding peptide (C6S‐p) on blocking CS functions were tested *in vitro* and *in vivo*. Results indicated that the expression of *CHPF* and *SDC1* was tightly associated within primary breast cancer tissue, and high expression of both genes exacerbated patient prognosis. Transforming growth factor beta (TGF‐β) signaling was implicated in the regulation of CHPF and SDC1 in breast cancer cells. CHPF supported CS–SDC1 stabilization on the cell surface, modulating macropinocytotic activity in breast cancer cells under nutrient‐deprived conditions. Furthermore, C6S‐p demonstrated the ability to bind CS‐SDC1, increase SDC1 degradation, suppress macropinocytosis of breast cancer cells, and inhibit tumor growth *in vivo*. Although other PGs may also be involved in CHPF‐regulated breast cancer malignancy, this study provides the first evidence that a CS synthase participates in the regulation of macropinocytosis in cancer cells by supporting SDC1 expression on cancer cells.

## Graphical abstract

This study reveals CHPF's role in breast cancer cell survival under nutrient deprivation, proposing a model where TGF‐β and CHPF increase cell surface SDC1, promoting macropinocytosis in cancer cells. The chondroitin sulfate‐specific binding peptide, C6S‐p, shows promise in counteracting CHPF effects by binding to CS‐SDC1, leading to SDC1 degradation and suppressing macropinocytosis.

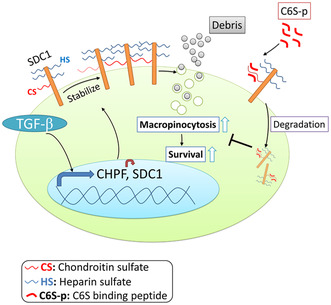

**Abbreviations**
C6S‐p: 6‐*O*‐sulfated chondroitin sulfate‐binding peptide
ChaseABC: chondroitinase ABC
CHPF: chondroitin polymerizing factor
CHX: cycloheximide
CS: chondroitin sulfate
CSPG: chondroitin sulfate proteoglycan
ECM: extracellular matrix
EIPA: 5‐[*N*‐ethyl‐*N*‐isopropyl] amiloride
FBS: fetal bovine serum
GAG: glycosaminoglycan
GalNAc: *N*‐acetylgalactosamine
HS: heparan sulfate
Hsase: heparinase
HSPG: heparan sulfate proteoglycan
PG: proteoglycan
scRNA‐seq: single‐cell RNA sequence
SDC1: syndecan 1
ssGSEA: single‐sample gene set enrichment analysis
TGF‐β: Transforming growth factor beta
WGA: wheat germ agglutinin

## 1 Introduction

Solid malignant tumors exhibit distinctive alterations in the tumor microenvironment, particularly in the composition of glycans, which play a pivotal role in shaping dynamic interactions between cancer cells and their surroundings [1, 2]. Among these glycans, glycosaminoglycans (GAGs) are major constituents in normal and cancer tissues, including chondroitin sulfate (CS), heparan sulfate (HS), keratan sulfate, and hyaluronan. Among these GAGs, hyaluronan exists as free chains, others are covalently linked to core proteins, forming proteoglycans such as chondroitin sulfate proteoglycan (CSPGs) and heparan sulfate proteoglycan (HSPGs) [3, 4]. Ectopically expressed CSPGs, such as CSPG4, versican, and decorin, have been identified as influential players in breast cancer progression [5, 6, 7, 8]. Besides, previous studies have highlighted the frequent detection of abnormal Oncofetal‐CS in breast cancer tissue [9]. Notably, chondroitin polymerizing factor (CHPF) has been identified as the main CS synthase in breast cancer tissue, significantly associated with the poor prognosis of breast cancer patients [10, 11, 12]. Despite these findings, the biological functions of abnormal CS and CSPGs, as well as their potential as druggable targets, remain largely unknown.

The biosynthesis of chondroitin sulfate (CS) chains initiates with *N*‐acetylgalactosamine (GalNAc) linking to a tetrasaccharide structure of protein backbone by CSGALNACT transferases. Subsequent polymerization is catalyzed by a group of bifunctional enzymes (CHSY1, CHPF, CHPF2, and CHSY3), which possess both β1–3 glucuronosyltransferase and β1–4 *N*‐acetylgalactosaminyltransferase activities [3, 4, 13]. Linearly polymerized CS chains comprise from few to over 50 disaccharide repeat units and undergo extensive modifications based on the spectrotemporal expression of CS epimerases and CS sulfotransferases. Concerning CS sulfation, the GalNAc residue is commonly *O*‐sulfated at C‐4 (4‐*O*‐sulfated CS; C4S) or C‐6 (6‐*O*‐sulfated CS; C6S) in mammary cells. Consequently, a single CS chain typically consists of a series of variably sulfated units, and the composition of CS on one CSPG may vary dramatically across different cell types.

Rapidly growing breast tumor tissue is well known for generating a stressful environment characterized by tissue stiffness, fibrosis, and poor vascularization, leading to nutrient scarcity. In response to this low‐nutrient stress, cancer cells are proposed to scavenge amino acids, carbohydrates, nucleic acids, and lipids from extracellular cell necrosis debris through macropinocytosis [14, 15]. This phenomenon has been observed in various tumor types, including pancreatic ductal adenocarcinoma and breast cancer. Beyond sustaining cancer cell survival in nutrient‐deprived tissues, this adaptive mechanism may confer resistance to nucleotide biosynthesis‐based targeted therapies [16, 17]. Given that proteoglycans exhibit broad binding affinity to numerous extracellular proteins, they have been shown to mediate various endocytosis pathways and exosome uptake [18, 19, 20]. Considering the crucial role of growth factor signaling in exosome uptake via macropinocytosis [14, 21], and the tight regulation of growth factor signaling by CSPGs, it is highly likely that changes in CS within cancer cells contribute to this cell survival phenotype.

Our prior research established a significant association between CHPF and poor survival in breast cancer patients, especially those with advanced TNM stages, and CHPF promotes aggressive breast cancer phenotypes *in vitro* and *in vivo* [12]. Consistent with our findings, several recent independent studies have also drawn similar conclusions [10, 11, 22, 23]. These results underscore the potential pivotal role of CHPF in regulating breast cancer malignancy. The primary objective of this study is to unravel the functional aspects of CHPF‐derived abnormal CS or CSPGs in breast cancer, particularly in a nutrient‐deprived environment. Additionally, we aim to design a potential method to block CS functions to suppress breast tumor progression. The outcomes of this investigation hold promising potential for the development of novel treatments benefiting breast cancer patients.

## 2 Materials and methods

### 2.1 Reagents

Full‐length Chpf cDNA‐pCMV6 plasmid was purchased from OriGene (MR216423; Rockville, MD, USA). Recombinant Heparinase I, II, and III (Catalog #: 7897‐GH, 6336‐GH, and 6145‐GH) were purchased from R&D Systems (Minneapolis, MN, USA). Antibody against Syndecan 1 (D4Y7H; Cell Signaling, Danvers, MA, USA) was used for western blots. Chondroitinase ABC and CCK8 reagent were purchased from Sigma‐Aldrich (St. Louis, MO, USA). 10 000 MW dextran conjugated with tetramethylrhodamine (D1817) and dextran conjugated with Cascade Blue (D1976) were purchased from Thermo Fisher Scientific Inc (Waltham, MA, USA). Breast cancer primary tissue array (BR10010e) was purchased from US Biomax, Inc (Rockville, MD, USA). Sequence of C6S blocking peptide (C6S‐p) was referring to the references [24, 25]. This N‐terminal biotinylated C6S‐p (Biotin‐EKRIWFPYRRF) and an identical amino acid composition scramble peptides (Biotin‐RPWREKIFYRF) were synthesized by Kelowna International Scientific Inc., New Taipei City, Taiwan. The peptides were purified by HPLC (> 98% in purity), and confirmed by mass spectrometry. TGFβR‐I inhibitor Ly364947 (#HY‐13462) was purchased from MedChem Express Co., Ltd (Monmouth Junction, NJ, USA).

### 2.2 Cell culture and transfection

MDA‐MB‐231 (RRID: CVCL_0062), HS578T (RRID: CVCL_0332), 4T1 (RRID: CVCL_0125), and Jurket (RRID: CVCL_0367) cell lines were authenticated using short tandem repeat profiling analysis in 2022. All cell lines were obtained from the American Type Culture Collection (Manassas, VA, USA) in 2014, and cultured in the complete medium which contains DMEM (Life Technologies, Waltham, MA, USA) with 0.1 mm sodium pyruvate, 10% FBS (5% FBS for 4T1 cells), 2 mm l‐glutamine, 100 IU·mL^−1^ penicillin, and 100 μg·mL^−1^ streptomycin. Plasmids were transfected to cultured cells using Lipofectamine™ 3000 (Thermo Fisher Scientific Inc.). The transfected cells were selected with 600 μg·mL^−1^ of G418. Lipofectamine RNAiMAX (Thermo Fisher Scientific Inc.) was used for siRNA transfection. ON‐TARGETplus SMARTpool siRNA against CHPF or Sdc1, and non‐targeting control were purchased from Dharmacon (Thermo Fisher Scientific Inc.). Twenty nanomoles of siRNA was used. All experiments were performed with mycoplasma‐free cells, which were routinely tested using PCR analysis.

### 2.3 Immunohistochemistry

Arrays were incubated with CHPF antibody (1 : 200) in 5% bovine serum albumin/PBS and 0.1% Triton X‐100 (Sigma, St. Louis, MO, USA) for 16 h at 4 °C. UltraVision Quanto Detection System (Thermo Fisher Scientific Inc.) was used to amplify primary antibody signal. The specific immunostaining was visualized with 3,3‐diaminobenzidine (DAB) and nuclear was stained by hematoxylin (Sigma).

### 2.4 Generate necrotic cell debris

The procedure of preparing necrotic cell debris from Jurkat cells was modified according to previous studies [26, 27]. Cultured Jurkat cells were suspended in PBS at 2 × 10^7^ cells·mL^−1^ and incubated at 56 °C in a water bath for 60 min. Dead cells were confirmed by trypan blue staining. Live and apoptotic cells were removed by low‐speed centrifuge (200 ***g***) for 5 min. The supernatant was transferred to a new tube, and the necrotic cell debris was collected by high‐speed centrifuge (8000 ***g***) for 15 min.

### 2.5 Macropinocytosis assays and image flow cytometry

For dextran uptake assays, breast cancer cells (2 × 10^4^) were seeded on coverside in 24 wells plate, and allowed to attach in complete culture medium. Low‐nutrient medium, no glucose DMEM (Cat. 11966025) added with 0.1% FBS, was replaced 16 h before adding dextran or necrotic debris. For dextran uptake assays, 10 000 MW Texas Red dextran (1 mg·mL^−1^; D1871; Invitrogen, Carlsbad, CA, USA) was added to the cell for 30 min, and washed three times with PBS. For confocal microscopy, cells were fixed with 4% paraformaldehyde in PBS for 30 min, and cell membrane was stained with FITC‐Wheat Germ Agglutinin (WGA; Vector Laboratories, Burlingame, CA, USA) for 60 min. Cell nuclei were labeled with Hoechst 33342 in PBS with 0.1% Triton X100 for 20 min.

For image flow cytometry, cells were resuspended by EDTA, and stained with APC anti‐human SDC1 antibody (DL‐101; BioLegend, San Diego, CA, USA) or APC anti‐mouse SDC1 (281‐2; BioLegend). All samples were analyzed with the Amnis ImageStreamX Mk II and inspire acquisition software (Merck KGaA, Darmstadt, Germany). The 40× magnification objective was employed, and at least 5 × 10^4^ events were collected for each sample. Data were analyzed and displayed with amnis ideas 6.2 analysis software (Merck KGaA). Internalization (macropinocytosis) capacity was measured as the mean number of particles per cell. Internalization index was determined employing the equation: [% macropinocytosis cells containing at least one particle] × [mean particle count per cell] [28, 29].

### 2.6 Confocal microscopy and macropinocytic index

Confocal images were captured by ZEISS LSM 980 confocal microscopy (Carl‐Zeiss, Oberkochen, Germany), and a 63× 1.4 NA Phase oil objective was used. Each confocal image of cell slides was stacked scanned images into one graphic. The total thickness in one capture field was 2.0 μm. The macropinocytic index was determined using the image j software (http://imagej.nih.gov/ij/) in accordance with a published protocol with slight modifications [30]. The process involved counting the number of particles and dividing it by the cell number in each field, to minimize the impact of varying cell densities. Three to five fields were measured for each group, and five independent experiments were carried out. This methodology ensures accurate and reliable results for the macropinocytic index.

### 2.7 Cell viability assay

Cell viability was assessed using the CCK8 assay. 2 × 10^3^ cells were seeded into 96‐well culture plates. A low‐nutrient medium (no glucose DMEM, 0.1% FBS) with or without necrotic cell debris (generated from 5 × 10^5^ Jurkat cells per well) was added and incubated for 48 h. CCK8 reagent was added for 4 h, and the absorbance of O.D. Four hundred and fifty nanometer was measured for each well. Experiments were repeated for four times independently and data were shown by relative fold changes.

### 2.8 Peptide treatment and peptide pull‐down assay

For macropinocytosis assay and cell viability assay, 50 μm of C6S‐p and scrambled peptide were used in culture cell for the indicated time. For the C6S‐p pulldown assay, a total of 0.8 mg cell lysate was mixed with C6S‐p gently and incubated at 4 °C for 16 h. Streptavidin beads (SA‐5010; Vector Laboratories) were added to the lysate and incubated for 3 h. The beads pulldown samples were analyzed by Western blotting.

### 2.9 Animal experiment

1 × 10^5^ of 4T1 cells were injected into the third mammary fat pad of 6‐week‐old female BALB/c mice (National Laboratory Animal Center, Tainan, Taiwan). The scrambled peptide or C6S‐p peptide was diluted in PBS to 0.3 μg·μL^−1^ and injected intratumorally at one‐third of the tumor volume as treatment. Lung metastasis was measured 3 weeks after the tumor was surgically removed. Mice were housed in individually ventilated cages within the Experimental Animal Center of Chung Shan Medical University. All animal experiments in this study were reviewed and approved by the Institutional Animal Care and Use Committee (IACUC) of Chung Shan Medical University Experimental Animal Center, Taiwan (Approval No: 2423).

### 2.10 Analysis of human breast cancer scRNA‐seq data

Processed scRNA‐seq data was downloaded from the GEO Series accession number GSE176078. Quality control, normalization, scaling, dimensionality reduction, and clustering of the data followed the standard Seurat workflow (version 4) [31] using default parameters. Cells were labeled with the original annotation. The single‐sample gene set enrichment analysis (ssGSEA) was performed using the escape package in R [32]. Plots were created using the dittoSeq package for both the single‐cell data and the ssGSEA results.

### 2.11 Statistical analysis

Data were analyzed by using graphpad prism 7 (Boston, MA, USA) and Microsoft Excel (Redmond, WA, USA). CHPF expression and clinic pathologic variables of breast cancer tissue array were analyzed by two‐tailed Fisher exact test. Survival curves were analyzed by Kaplan–Meier analysis and the log‐rank test. When *P* value < 0.05, it is defined as statistically significant.

## 3 Results

### 3.1 Co‐expression of *CHPF* and *SDC1* in primary breast cancer tissue predicts poor survival and strong cell‐ECM signaling

**Fig. 1:**
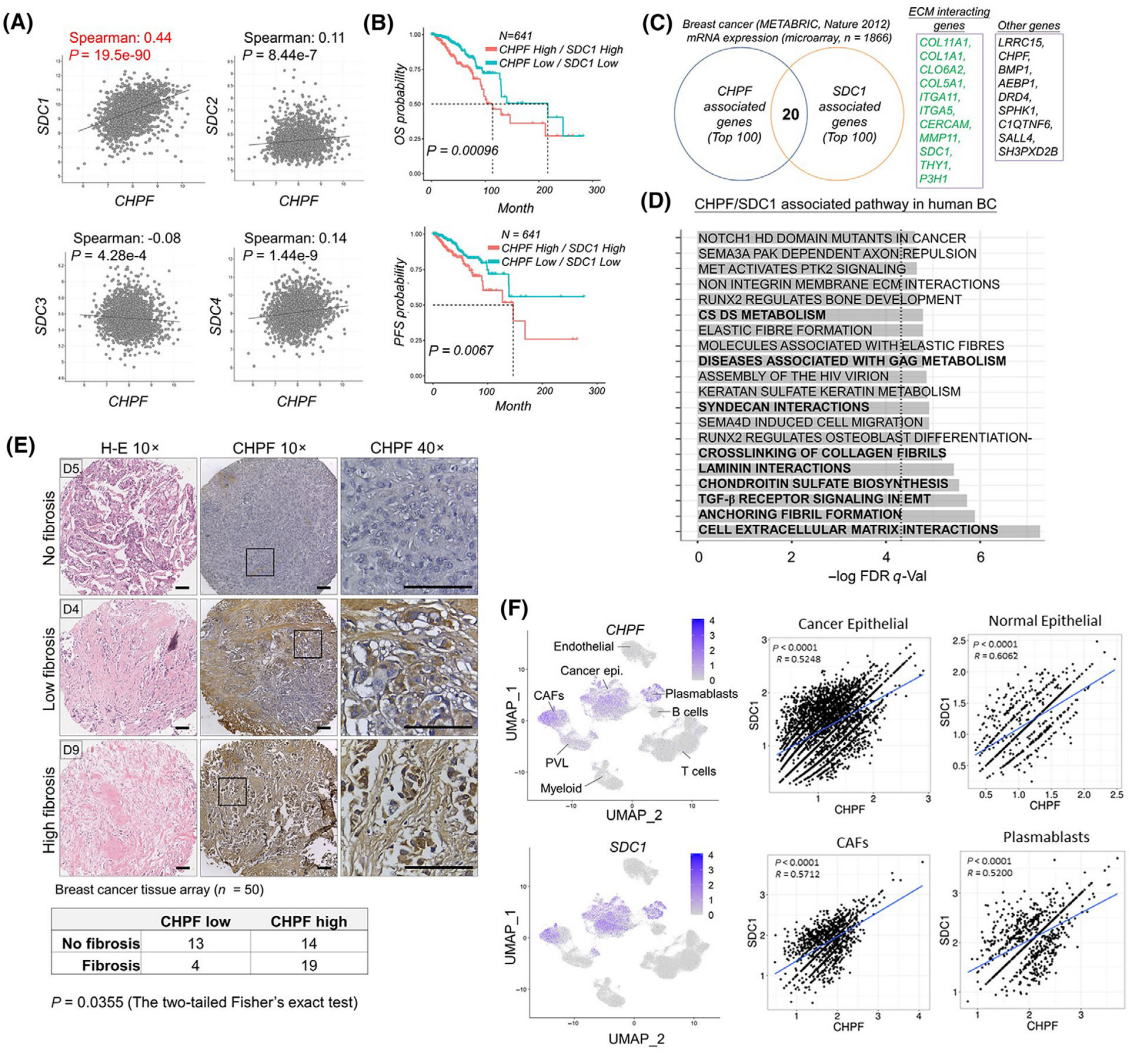
Expression of *CHPF* associated with *SDC1* predicts poor prognosis of patients with breast cancer. (A) Correlation of gene expression of *CHPF* and syndecans (*SDC1*, *SDC2*, *SDC3*, and *SDC4*) in human breast cancer tissues. (B) Kaplan Meier analysis of overall survival (OS) and progress free survival (PFS) of breast cancer patients with high expression of both *CHPF* and *SDC1* and low expression of both *CHPF* and *SDC1*. (C) Overlapping of CHPF and SDC1 positive associated genes in METABRIC‐breast invasive carcinoma dataset. ECM, extracellular matrix. (D) Pathway enrichment analysis using the Reactome knowledgebase. Bold text indicates the pathways involved in GAG formation and extracellular matrix interactions. (E) Representative images of H&E stain and immunohistochemistry on breast cancer tissue array (*n* = 50; case number: D4, D5, and D9 are shown) using anti‐CHPF antibody. Tissues were counterstained with hematoxylin. Amplified images were shown at right. Scale bar = 150 μm. (F) Analysis of cellular expression levels of CHPF and SDC1 using a single‐cell RNA‐sequencing dataset (scRNA‐seq) [37] with 26 primary breast cancer tissues. Smart‐seq2 data were downloaded with cell type annotations. CAFs, cancer‐associated fibroblasts; PVL, perivascular‐like cells.

Mega analysis of cancer genomics revealed that CS biosynthesis genes tend to cluster with specific proteoglycans (PGs) in a cancer type‐dependent manner [33], and several recent studies proposed that CHPF is a crucial CS biosynthesis that upregulated in breast cancer tissue and associated with poor prognosis [10, 11, 12]. However, the influences of CHPF‐modulated PGs in cancer progression are still not well understood. We first examined *CHPF*‐associated genes in the METABRIC‐breast invasive carcinoma dataset (*n* = 1866) using cBioPortal [34]. Data revealed that syndecan‐1 (*SDC1*), a HSPG that also carries one or two CS chains [35], is the most significant PG that is positively associated with *CHPF* (Fig. 1A). Meanwhile, the expression of both *CHPF* and *SDC1* are also positively correlated with the expression of C6S sulfotransferase (*CHST3*) and C4S sulfotransferase (*CHST11*) in this breast cancer dataset (Fig. S1A). Similar results were also observed in the TCGA‐breast cancer dataset (*n* = 960) (data not shown). Moreover, TCGA‐breast cancer patients with high expression of both *CHPF* and *SDC1* (H/H) were significantly associated with shorter PFS and OS compared to the low expression (L/L) group (Fig. 1B), whereas the statistical differences were more significant than using *CHPF* or *SDC1* alone, and the shortest mean survival was observed in the H/H group (Fig. S1B,C). We observed that a group of well‐known ECM interaction genes, such as *COL11A1*, *COL1A1*, *CLO6A2*, *COL5A1*, *ITGA11*, *ITGA5*, and *MMP11*, were listed in the top 100 positively associated genes with both *CHPF* and *SDC1* in the METABRIC‐breast invasive carcinoma dataset (Fig. 1C). Pathway enrichment analysis using the Reactome knowledgebase [36] revealed several pathways significantly (FDR *q*‐Val < 0.05) enriched in the differentially expressed genes between two subsets of breast cancer patients (H/H vs. L/L), including cell extracellular matrix (ECM) interaction, TGFβ receptor signaling, chondroitin sulfate biosynthesis, and crosslink of collagen fibrils (Fig. 1D). In addition, through immunohistochemistry of breast cancer tissue array (*n* = 50), we noticed that local tissue fibrosis (over 20% of the section area was occupied by hyalinized collagen fibers) was positively associated with strong CHPF staining in cancer cells (Fig. 1E). To verify the association of *CHPF* and *SDC1* in specific cell types of tumor tissue, we leveraged a public single‐cell RNA sequence (scRNA‐seq) resource of 26 primary breast tumors [37]. Results indicated that the positive association of *CHPF* and *SDC1* was significant in cancer epithelium, myeloid cells, plasmablasts (B cells), and cancer‐associated fibroblasts (CAFs) in cancer tissue (Fig. 1F and Fig. S1A,B), suggesting common regulations between *CHPF* and *SDC1* may exist in different cell types.

### 3.2 Regulation between CHPF and SDC1 expression in breast cancer cells

**Fig. 2:**
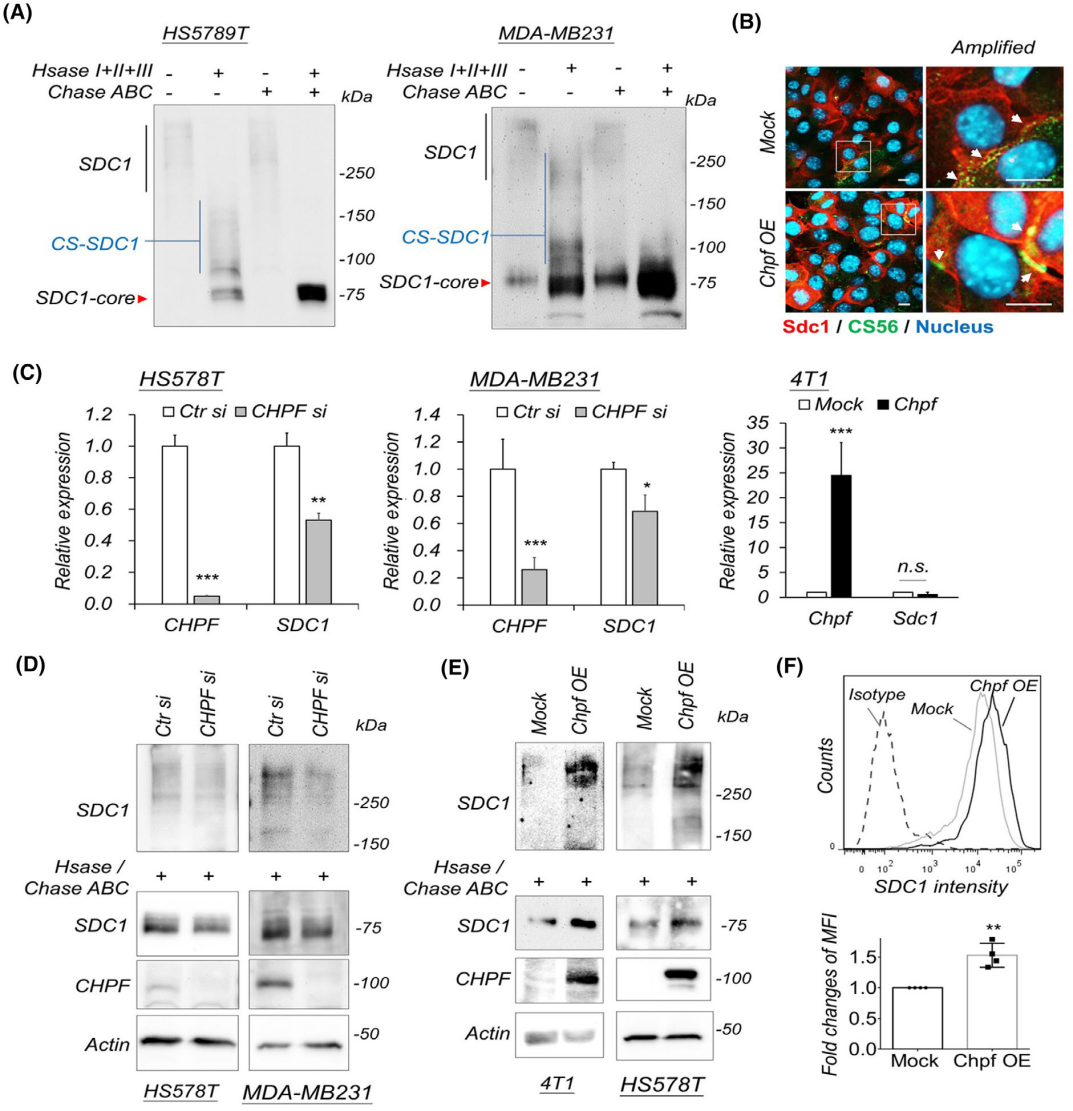
CHPF modulates SDC1 expression. (A) Western blots of SDC1 in breast cancer cells. Lysates were treated with heparinase (Hsase I + II + III) and/or chondroitin ABC (ChaseABC). Chondroitin sulfate SDC1 (CS‐SDC1) and GAG removed SDC1 core protein (SDC1‐core) were indicated. Representative images were shown from four independent experiments. (B) Immunofluorescence of SDC1 and CS56 in Chpf overexpressed (Chpf OE) and mock transfected (empty vector) 4T1 cells. White arrow indicates CS‐SDC1 on the cell surface. Scale bar = 10 μm. Representative images were shown from four independent experiments. (C) q‐PCR of *CHPF* and *SDC1* in *CHPF* siRNA silenced or overexpressed cells. Expression was normalized to *18S*, and relative expression to control were shown. *CHPF si*, *CHPF*‐specific siRNA transfected cells; *Ctr si*, control siRNA transfected cells. Mean ± SD was shown. *P < 0.05; **P < 0.01; ***P < 0.001 by two side *t*‐test. Representative data were shown from four independent experiments with similar results. (D, E) Western blots of CHPF and SDC1 in *CHPF* siRNA silenced or overexpressed cells. β‐Actin (Actin) was taken as loading control. Representative images were shown from four independent experiments. (F) Flow cytometry revealed cell surface SDC1 after overexpression of Chpf in 4T1 cells. Mean ± SD was shown. **P < 0.01 by two side *t*‐test. All experiments were repeated four times, and representative images were shown.

Given that co‐expression of *CHPF* and *SDC1* correlates with poor prognosis of breast cancer, we next aim to examine the regulation between CHPF and SDC1. The results from western blots of breast cancer cell lines indicate that SDC1 is extensively glycosylated, with a molecular weight exceeding 150 kDa. Further analysis using heparinase and/or chondroitin ABC on protein lysate confirmed that a significant portion of SDC1 in breast cancer cells is associated with CS chains (Fig. 2A). Additionally, the transient knockdown of *Sdc1* by siRNA confirmed the specificity of the anti‐SDC1 antibody (Fig. S2A). It is noteworthy that the anti‐SDC1 antibody appears to have much better affinity on western blots when GAG chains were enzymatically removed. Double immunostaining of SDC1 with an anti‐C6S antibody (CS56) in breast cancer cells revealed that the SDC1 mainly appeared on cell surface (Fig. 2B). In addition, overexpression of CHPF showed a trend in increasing both SDC1 and SDC1/CS56 co‐localization. In mRNA levels, we transiently silenced or overexpressed *CHPF* in breast cancer cell lines to investigate its regulation. qPCR showed that *CHPF* silencing mildly decreased *SDC1* expression, while overexpression of *CHPF* had no influence on *SDC1* expression (Fig. 2C), implying that endogenous *CHPF* may help maintain the expression of *SDC1*. In contrast, overexpression or siRNA silencing of *SDC1* had no effects on *CHPF* expression (Fig. S2B). In the protein levels, western blots indicated that silencing of CHPF decreased glycosylated SDC1 (Fig. 2D), and overexpression of CHPF significantly increased glycosylated SDC1 in breast cancer cells and HEK293 cells (Fig. 2E and Fig. S2C), similar results were observed when GAG chains were enzymatically removed. Additionally, flow cytometry also showed that cell surface SDC1 was increased on CHPF overexpressed cells (Fig. 2F). Examining shedding SDC1 in culture medium revealed no obvious changes (Fig. S2D). Collectively, these data suggested that CHPF‐mediated CS formation could support SDC1 accumulation on the cell surface.

### 3.3 TGF‐β signal regulates both CHPF and SDC1 expression in breast cancer cells

**Fig. 3:**
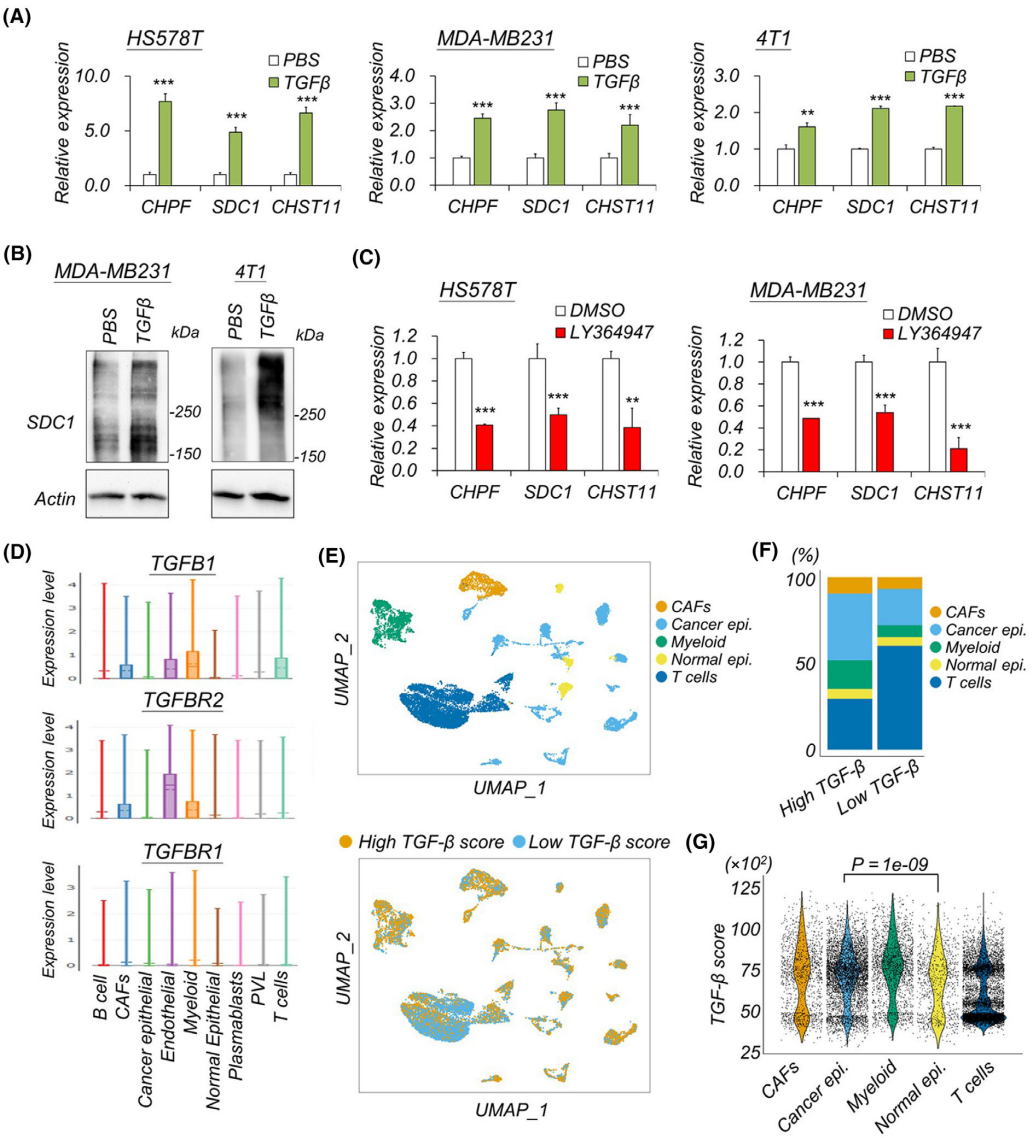
TGF‐β signal regulates both CHPF and SDC1 expression. (A) q‐PCR of *CHPF*, *SDC1*, and *CHST11* in TGF‐β treated cells. Mean ± SD was shown. **P < 0.01; ***P < 0.001 by two side *t*‐test. Representative data were shown from three independent experiments with similar results. (B) Protein levels of SDC1 after TGF‐β treatment. Representative images were shown from four independent experiments. (C) q‐PCR of *CHPF*, *SDC1*, and *CHST11* in TGF‐β type‐I receptor inhibitor, LY364947, treated cells. Mean ± SD was shown. **P < 0.01; ***P < 0.001 by two side *t*‐test. Representative data were shown from three independent experiments with similar results. (D) Expression level of *TGFB1*, *TGFBR1*, and *TGFBR2* in major cell types of primary breast cancer tissue. Data generated by single‐cell portal (https://singlecell.broadinstitute.org/single_cell) using dataset from Wu et al. [37]. Note that the gene expression levels were the corrected counts transformed using LogNormalize function in seurat. (E) Top, overview of the reanalysis of GSE176078 scRNA‐seq dataset. Bottom, UMAP plot of cells colored by high or low TGF‐β groups, calculated by ssGSEA. (F) Percentage composition of cell types for high or low TGF‐β signaling groups. (G) Violin and jitter plot of TGF‐β score across major cell types in breast cancer tissue. The *P* value is calculated by a two‐sample Wilcoxon test. CAFs, cancer‐associated fibroblasts; epi., epithelium; PVL, perivascular‐like cells.

Because of the predicted association of TGF‐β receptor signaling with CHPF/SDC1 in human breast cancer (Fig. 1D), a pathway known for its dominance in promoting cancer tissue fibrosis [38, 39], we investigated the impact of TGF‐β signaling on the expression of CHPF and SDC1. Results demonstrated a significant increase in both mRNA and protein levels of CHPF and SDC1 after the addition of TGF‐β to the culture medium for 48 h (Fig. 3A,B). As a positive control for TGF‐β signaling, we measured chondroitin 4‐*O*‐sulfotransferase 11 (*CHST11*), known to be regulated by TGF‐β [40, 41]. Additionally, the use of the TGF‐β type‐I receptor inhibitor, LY364947, in cultured cells significantly suppressed the expression of both *CHPF* and *SDC1* (Fig. 3C). Considering the multifunctionality of TGF‐β, and its often upregulation in the tumor microenvironment, we aimed to identify the major TGF‐β‐responding cell types in breast cancer tissue. Reanalysis of a human breast cancer single‐cell RNA‐seq dataset (GSEA176078) [37] revealed the expression of TGF‐β and TGF‐β receptors in cancer cells, myeloid cells, cancer‐associated fibroblasts (CAFs), and normal endothelial cells (Fig. 3D). To estimate the TGF‐β signaling score, we employed single‐sample gene set enrichment analysis (ssGSEA) of a 6‐gene TGF‐β signature (SLC20A1, XIAP, TGFBR1, BMPR2, FKBP1A, and SKIL) (Fig. 3E) [42]. The results indicated that cancer epithelial cell was the dominant cell type in the high TGF‐β score group (Fig. 3F). The TGF‐β scores revealed a very low but significantly positive correlation with the expression of CHPF and SDC1 (Fig. S3). Moreover, cancer epithelial cells exhibited a significantly higher TGF‐β score than normal epithelial cells (Fig. 3G). Collectively, these data suggest that TGF‐β signaling may serve as a common upstream regulator for CHPF and SDC1 in breast cancer cells, particularly in the context of cancer epithelial cells.

### 3.4 CHPF mediates macropinocytosis and enhances breast cancer cell survival in low‐nutrient conditions

**Fig. 4:**
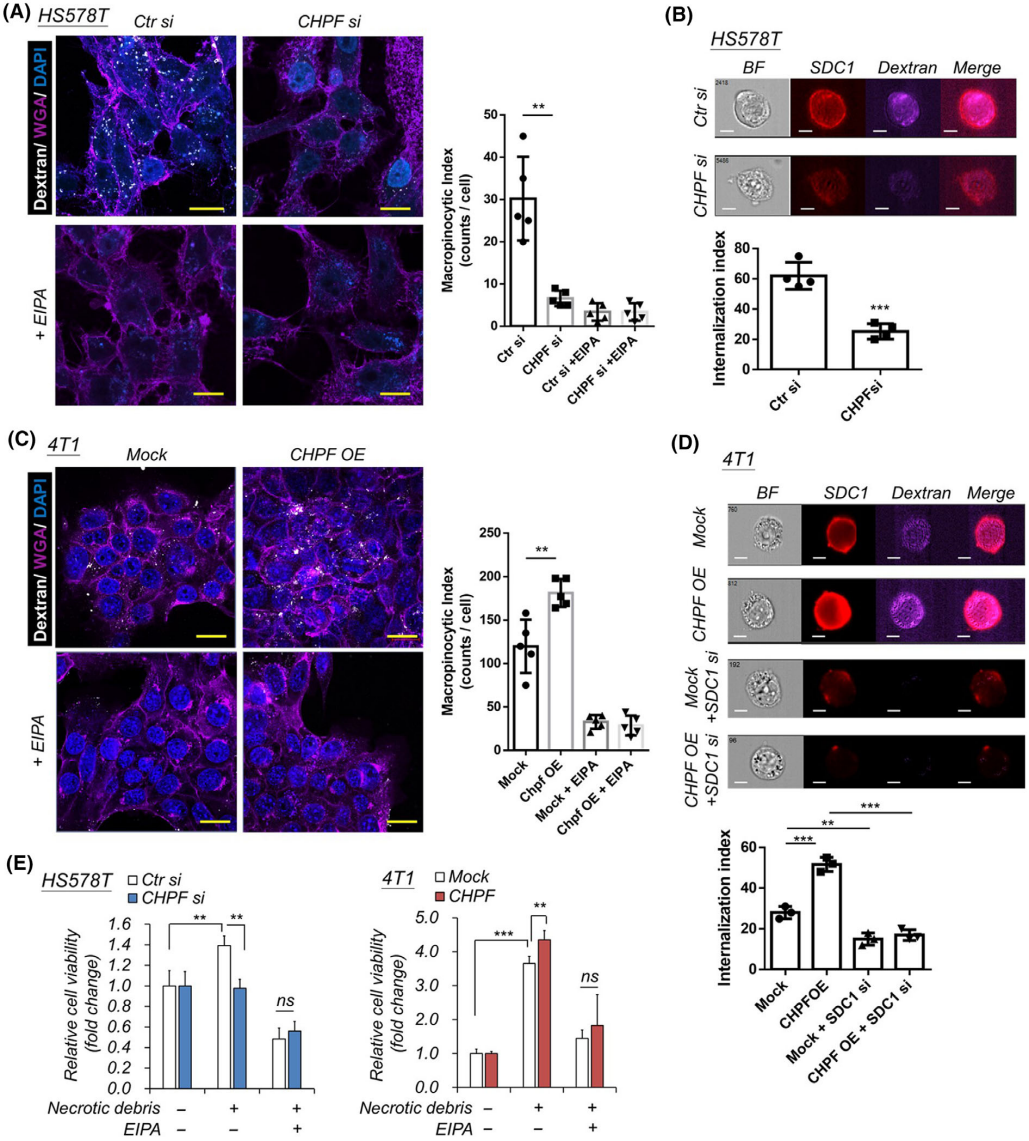
CHPF modulates macropinocytosis of breast cancer cells. (A) Silencing of CHPF suppressed macropinocytosis in low‐nutrient culture conditions. The uptake of dextran in HS578T cells displayed as white dots. Wheat germ agglutinin (WGA) was used to label cell membranes to outline the cell boundary (purple). EIPA was used as a macropinocytosis suppressor. *CHPF si*, *CHPF*‐specific siRNA transfected cells; *Ctr si*, control siRNA transfected cells. Mean ± SD was shown from five independent experiments (right). **P < 0.01; by two side *t*‐test. Scale bar 20 μm. (B) Measuring dextran internalization using imaging flow cytometry. Representative images were shown at top. Scale bar 7 μm. Mean ± SD was shown from four independent experiments. ***P < 0.001 by two side *t*‐test. (C) Overexpression of CHPF (CHPF OE) increased macropinocytosis in 4T1 cells. Mean ± SD was shown from five independent experiments (right). **P < 0.01; by two side *t*‐test. Scale bar 20 μm. (D) Imaging flow cytometry of dextran internalization in CHPF overexpressed cells and SDC1‐silenced cells. Mean ± SD was shown from three independent experiments. Representative images were shown at top. Scale bar 7 μm. **P < 0.01; ***P < 0.001 by two side *t*‐test. (E) Fold change of breast cancer cell viability in low‐nutrient culture condition feed with or without cell debris. Mean ± SD was shown from three independent experiments. **P < 0.01; ***P < 0.001 by two side *t*‐test. ns, not significant.

Macropinocytosis is a highly‐conserved non‐selective endocytic process that has been shown to enhance tumor cells to uptake extracellular nutrients and cell debris, and enable tumors to offset starvation in nutrient‐deprived conditions [16, 17]. Recent reports indicated that cell surface SDC1 triggers cellular signals that control macropinocytosis in pancreatic cancer cells [15, 43]. Given that CHPF modulates SDC1 expression, we thus examined whether macropinocytosis was affected in breast cancer cells. Using receptor‐independent internalization of Texas Red‐labeled dextran uptake to visualize macropinosomes, we observed that silencing CHPF significantly decreased macropinocytic ability in low‐nutrient conditions in HS578T cells. Dextran uptake was inhibited when treating cells with EIPA (5‐[*N*‐ethyl‐*N*‐isopropyl] amiloride), a Na^+^/H^+^ exchanger inhibitor for transiently blocking macropinocytosis (Fig. 4A). Imaging flow cytometry of HS578T cells further confirmed that the internalized dextran and surface SDC1 were decreased when CHPF was silenced (Fig. 4B). Conversely, the overexpression of CHPF in 4T1 cells significantly enhanced macropinocytosis, as observed through confocal microscopy (Fig. 4C) and imaging flow cytometry (Fig. 4D). Furthermore, the internalization of dextran was significantly inhibited in SDC1‐siRNA silenced cells. To evaluate the impact of macropinocytosis on breast cancer cell survival in low‐nutrient culture conditions, necrotic debris from Jurkat cells was added to nutrient‐deprived medium. The results indicated that the addition of necrotic debris significantly enhanced cell viability. However, silencing CHPF suppressed this effect. Conversely, overexpression of CHPF further promoted necrotic debris‐enhanced cell viability (Fig. 4E). Additionally, the use of EIPA to inhibit macropinocytosis resulted in the suppression of necrotic debris‐induced cell viability.

### 3.5 CS‐binding peptide downregulates SDC1 protein and suppresses macropinocytosis

**Fig. 5:**
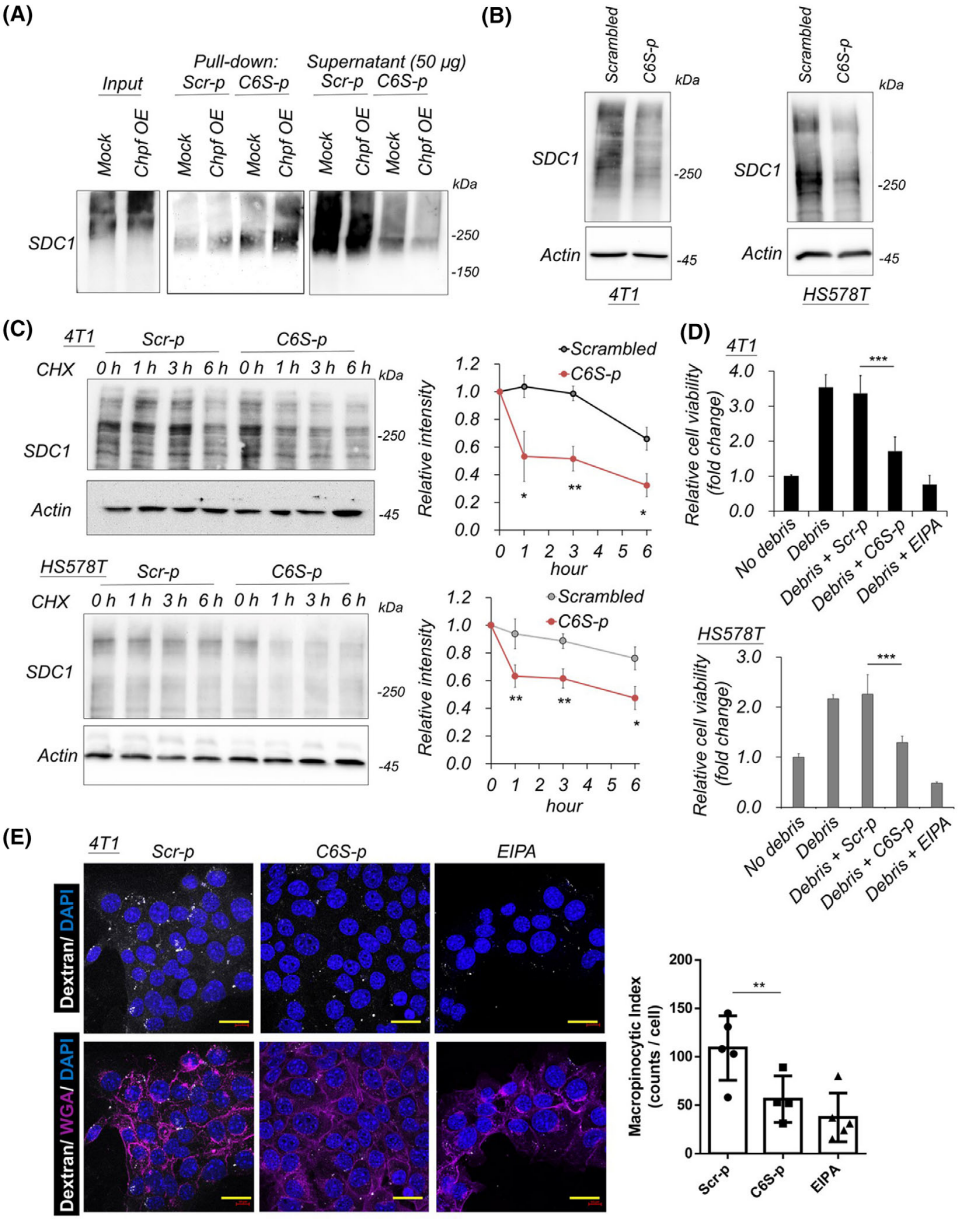
Chondroitin sulfate‐binding peptide enhances SDC1 degradation and suppresses macropinocytosis. (A) 6‐*O*‐sulfated chondroitin sulfate‐binding peptide (C6S‐p) pull‐down assay of protein lysate from mock (empty vector) and CHPF overexpressed (CHPF OE) cells. Scrambled peptide (Scr‐p) with an identical amino acid composition to C6S‐p was used as control. Representative images were shown from three independent experiments. (B) Breast cancer cells were treated with peptide for 48 h and analyzed by western blots. β‐Actin (Actin) was taken as loading control. Representative images were shown from four independent experiments. (C) Western blotting of SDC1 degradation. 4T1 cells or HS578T cells were treated with peptides and 20 μm cycloheximide (CHX) for 1, 3, and 6 h. Actin was taken as loading control. The relative protein levels were calculated from three independent experiments and shown on the right. Mean ± SD was shown. *P < 0.05; **P < 0.01 by two side *t*‐test. (D) C6S‐p suppresses cell debris‐enhanced cell viability. Mean ± SD was shown from three independent experiments. ***P < 0.001 by two side *t*‐test. (E) C6S‐p inhibits macropinocytosis in 4T1 cells. Cells were pretreated with Scr‐p or C6S‐p for 1 h, and dextran was added for uptake for 30 min. Wheat germ agglutinin (WGA) was used to label cell membranes (purple). EIPA was used as a macropinocytosis suppressor. Mean ± SD was shown from five independent experiments (right). **P < 0.01; by two side *t*‐test. Scale bar 20 μm.

Prior studies have shown that CHPF increases C6S on cancer cells [12, 44], and the C6S‐specific binding peptide (C6S‐p) identified from a phage display peptide library has been proposed to block certain C6S biological functions, such as rescuing neurite outgrowth and reducing glioma cell invasion [24, 25, 45, 46]. Thus, we investigated whether C6S‐p could target CS‐SDC1 on breast cancer cells. The protein pulldown assay using biotinylated C6S‐p revealed that glycosylated SDC1 binds to C6S‐p, with the affinity further increased by overexpression of CHPF (Fig. 5A). Subsequently, we tested the effects of treating breast cancer cells with C6S‐p. However, in complete culture medium (10% FBS in DMEM), treatment with C6S‐p (10–50 μm) showed no significant effects on cell viability and mobility (data not shown). In these experiments, the protein levels of SDC1 were notably decreased after 48 h of C6S‐p treatment in all tested breast cancer cell lines (Fig. 5B). To examine whether C6S‐p promotes SDC1 degradation, the protein synthesis inhibitor cycloheximide (CHX) was used to block protein translation, and the protein levels of SDC1 were measured. The results indicated that treatment with C6S‐p significantly accelerated SDC1 degradation in breast cancer cells compared to cells treated with the scrambled peptide (Fig. 5C). Additionally, C6S‐p treatment suppressed the necrotic debris‐promoted cell viability in a low‐nutrient culture condition (Fig. 5D), and the dextran uptake assay showed that C6S‐p significantly inhibited macropinocytic ability in breast cancer cells (Fig. 5E and Fig. S4). Collectively, these data suggest that the C6S‐binding peptide targets SDC1 and enhances SDC1 degradation, potentially suppressing macropinocytosis‐induced cell survival in a nutrient‐deprived microenvironment.

### 3.6 CS‐binding peptide treatment suppresses breast tumor growth and metastasis *in vivo*

**Fig. 6:**
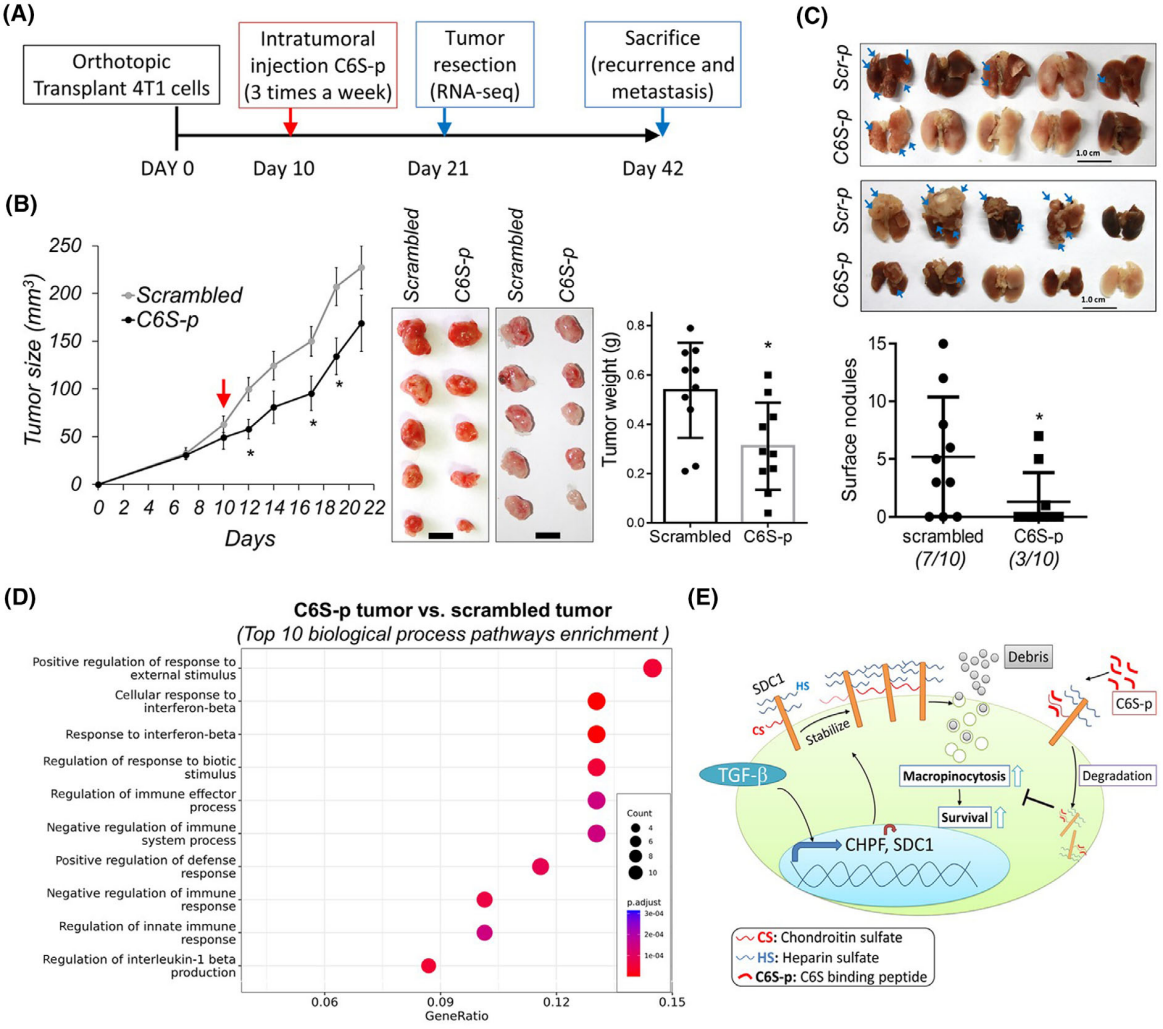
6‐*O*‐sulfated chondroitin sulfate‐binding peptide (C6S‐p) inhibits tumor progression *in vivo*. (A) Protocol of 4T1 tumor model and peptide treatments. (B) Measurement of tumor size and weight. Red arrow indicated the time point of peptide treatment. Tumor mass was surgically removed on day 21. Mean ± SD was shown. *P < 0.05; by two side *t*‐test. Scale bar 1 cm. Ten mice for each group. (C) Spontaneous cancer metastasis to lung. Blue arrow indicates tumor nodules. Incidence of metastasis was shown at bottom. Mean ± SD was shown. *P < 0.05; by two side *t*‐test. Ten mice for each group. Scale bar 1 cm. (D) Pathway enrichment analysis of differential expressed genes between C6S‐p and Scr‐p treated tumor tissue. (E) A proposed model illustrating that TGF‐β and CHPF enhanced cell surface SDC1 and promoted macropinocytosis of breast cancer cells. This model also demonstrated that C6S‐p promoted the degradation of SDC1 and suppressed cancer cells uptake cell debris for surviving in low‐nutrient microenvironment.

To further assess the impact of C6S‐p treatment in an animal tumor model, we employed an intratumor injection protocol of C6S‐p or a scrambled peptide in an established orthotopic 4T1 breast cancer tumor (Fig. 6A). Results indicated that administration of C6S‐p three times a week significantly suppressed tumor growth (Fig. 6B). After surgically removing the tumors, spontaneous cancer metastasis to the lung also significantly decreased in the C6S‐p treatment group (Fig. 6C). Pathway analysis of differentially expressed genes revealed that C6S‐p treatment induced a better innate immune response in tumor tissue, and IFN‐β and IL‐1β signals may also be enhanced (Fig. 6D). These data suggest an anti‐tumor effect of C6S‐p treatment *in vivo*.

## 4 Discussion

In this study, we established a strong association between the expression of *CHPF* and *SDC1* within primary breast cancer tissue. Notably, results heightened expression of both *CHPF* and *SDC1* positively correlated with exacerbating patient prognosis. Given cancer tissue fibrosis or radiotherapy‐induced tissue fibrosis are directly controlled by TGF‐β signaling [38, 47], we revealed that added TGF‐β stimulated expression of CHPF and SDC1 in cultured breast cancer cells, offering insights into the link between CHPF and SDC1 across diverse cell types in cancer tissue. We further indicated that CHPF may support CS‐SDC1 stabilization on cell surface, endorsing heightened macropinocytotic activity in breast cancer cells under nutrient‐deprived conditions. The administration of a CS‐specific binding peptide, C6S‐p, emerges as a potential strategy to counteract the effects induced by CHPF. C6S‐p may bind to CS‐SDC1, and lead to promoting SDC1 degradation, subsequent suppression of macropinocytosis in breast cancer cells (Fig. 6E). C6S‐p also inhibited tumor growth *in vivo*. Although this study primarily focuses on the involvement of CHPF and SDC1 in breast tissue, it is important to acknowledge that other CSPGs may also be regulated by CHPF, and contribute to cancer malignancy. Notably, our study marks the initial evidence of a CS synthase participating in the regulation of macropinocytosis in cancer cells through the modulation of SDC1.

Previous investigations have highlighted aberrant expression of CS synthases and sulfotransferases linked to malignant phenotypes in breast cancer. For instance, the CS sulfotransferases, CHST11 and CHST15, have been implicated in promoting invasiveness and metastasis of breast cancer cells [48, 49]. Excessive CS accumulation in malignant breast cancer tissue is associated with an adverse patient outcome [50]. Taking advantage of the increasing number of RNA sequence data of cancer patients released in recent years, we and other groups confirm the CHPF is the critical CS synthase in advanced breast cancer [9, 10, 11, 12]. Intriguingly, our study identifies a group of collagen subtypes co‐expressed with CHPF in breast cancer tissue, revealing a potential link between CS accumulation and tissue fibrosis. Immunohistochemistry on a breast cancer tissue array further confirms excessive collagen, tissue fibrosis, and CHPF expression. This novel finding suggests a possible involvement of CS accumulation in tissue fibrosis, although further investigations are warranted to elucidate whether CHPF‐related breast tissue fibrosis occurs early in tumorigenesis or as a consequence of cancer treatments such as radiotherapy and chemotherapy.

Our data revealed the significant association between *CHPF* and *SDC1* in bulk RNAseq of breast cancer tissue as well as individual major cell types using scRNAseq data. Further searching the correlation of *CHPF* and *SDC1* in other types of cancer using cBioPortal [34], we found that this correlation is not universal, which revealed only mild or no correlation between these two genes in lung cancer, cervical cancer, and other carcinoma (data not shown). We assumed this variation may raise from the tissue specific CS synthases context or the differential regulation of TGF‐β signaling. Referring to the scRNAseq of breast cancer (Fig. 3D), the myeloid cells are one the main source of TGF‐β production in primary tumor tissue. Our previous study demonstrated that up‐regulation of CHPF increased the G‐CSF in tumor tissue, which increased the number of myeloid‐derived suppressor cells (MDSC) in a breast cancer model [12], potentially leading to further increases of TGF‐β signaling intensity in breast cancer tissue. In addition to the evidence presented in this study suggesting that TGF‐β signaling regulates CHPF and SDC1 expression in breast cancer cells, the *CHPF* knockdown experiments also resulted in a partial decrease in *SDC1* gene expression levels (Fig. 2C), indicating that other complex regulatory mechanisms may still remain undiscovered.

Current evidence pointed that SDC1 contains five potential GAG attached sites, with three for HS chains and two for CS chains [35]. However, the precise composition may depend on the cell types. In this study, we demonstrate the presence of CS‐SDC1 in our tested breast cancer cells, supported by western blots when HS chains were removed, as well as colocalization with CS56 staining (Fig. 2A,B). Among the four members of syndecans, SDC1 is the most studied in tumorigenesis. Several reports have highlighted the critical role of SDC1 in promoting breast cancer malignancy, significantly associated with a worse prognosis for patients [51]. In addition, the up‐regulation of SDC1 in breast cancer stromal tissue has been proposed to promote cancer cell mobility and metastasis in mouse models [52, 53]. Previous reports have suggested that HS chains are more important for optimal SDC1 function. HS chains of SDC1 can serve as a coreceptor for various growth factors, facilitating their binding to corresponding receptors and even supporting exosome uptake [54, 55, 56]. In contrast, the function of CS chains on SDC1 is less investigated. We observed that the expression of CHPF regulates SDC1 protein levels in breast cancer cells, suggesting that increased CS supports the stability of SDC1 on the cell membrane.

The immunoreactivity of the anti‐CS antibody used in this study (CS56) requires CHST3‐mediated C6S structure [57]. Our study and a previous report indicated C6S may have crucial functions in regulating breast cancer malignancy [12, 50]. However, only a few studies have examined the methods for blocking C6S bioactivities in cancer treatment. In a previous study, a C6S‐specific binding peptide was used on cultured glioblastoma cells, and the treatment suppressed glioma cell mobility accompanied by promoting CD44 degradation, but its effects were not evaluated *in vivo* [45]. Dr Karumbaiah reported that Surfen could block extratumoral CS and inhibit the invasion of F98 (a rat GBM cell line) cells, but the therapeutic effects of Surfen are not significant in animal models [58]. In this study, we employed intratumoral injections of C6S‐p to effectively inhibit tumor growth and metastasis. Several HSPGs and CSPGs, including SDC1, SDC4, versican, CSPG4, and CD44, were reported to be up‐regulated in breast cancer tissue. All these proteoglycans that carry CS chains on both cancer cells and stromal cells are potential targets of C6S‐p, contributing to tumor suppression. Therefore, the effects of C6S‐p on inducing SDC1 degradation and inhibiting macropinocytosis should be only one of the mechanisms of C6S‐p *in vivo*.

## 5 Conclusions

Our study uncovers a novel function of CHPF in supporting tumor cell survival. CHPF modulates the expression of SDC1 and regulates macropinocytosis in nutrient‐deprived conditions. Furthermore, our study proposes the use of C6S‐p to block CHPF‐mediated biological functions, motivating further studies aimed at optimizing C6S‐p therapy protocols in breast cancer models.

## Conflict of interest

The authors declare no conflict of interest.

## Author contributions

C‐HL conceived and supervised the project. C‐HL, H‐RY, and W‐CL designed the project. C‐HL, H‐RY, W‐CL, C‐HC, Y‐AS, Y‐WH, and P‐KS contributed to the experimental design. C‐HC, Y‐AS, Y‐WH, CH, Y‐HC, and Y‐LC performed experiments. H‐RY and W‐CL performed the histological analyses. C‐HC and CH analyzed public databases. C‐HL, H‐RY, and W‐CL prepared the manuscript. All authors read the manuscript, provided feedback, and approved the final manuscript.

### Peer review

The peer review history for this article is available at https://www.webofscience.com/api/gateway/wos/peer‐review/10.1002/1878‐0261.13667.

## Supporting information


**Fig. S1.** Association of CHPF and SDC1 in breast cancer tissue.
**Fig. S2.** CHPF regulates SDC1 expression.
**Fig. S3.** Correlation between TGF‐β score and expression of *CHPF* or *SDC1*.
**Fig. S4.** CS‐binding peptide suppresses macropinocytosis in HS578T cell.

## Data Availability

Processed scRNA‐seq data was downloaded from the GEO Series accession number GSE176078 (https://www.ncbi.nlm.nih.gov/geo).
