# Supplementary material for: Targeting chondroitin sulfate suppresses macropinocytosis of breast cancer cells by modulating syndecan‐1 expression
Source: Mol Oncol. 2024 May 21;18(10):2569–85. doi: 10.1002/1878-0261.13667 (PMC11459036; doi:10.1002/1878-0261.13667)
Supplement: Supplementary file 1 — Fig. S1. Association of CHPF and SDC1 in breast cancer tissue. Fig. S2. CHPF regulates SDC1 expression. Fig. S3. Correlation between TGF‐β score and expression of CHPF or SDC1. Fig. S4. CS‐binding peptide suppresses macropinocytosis in HS578T cell. [file MOL2-18-2569-s001.pdf]

Figure S1

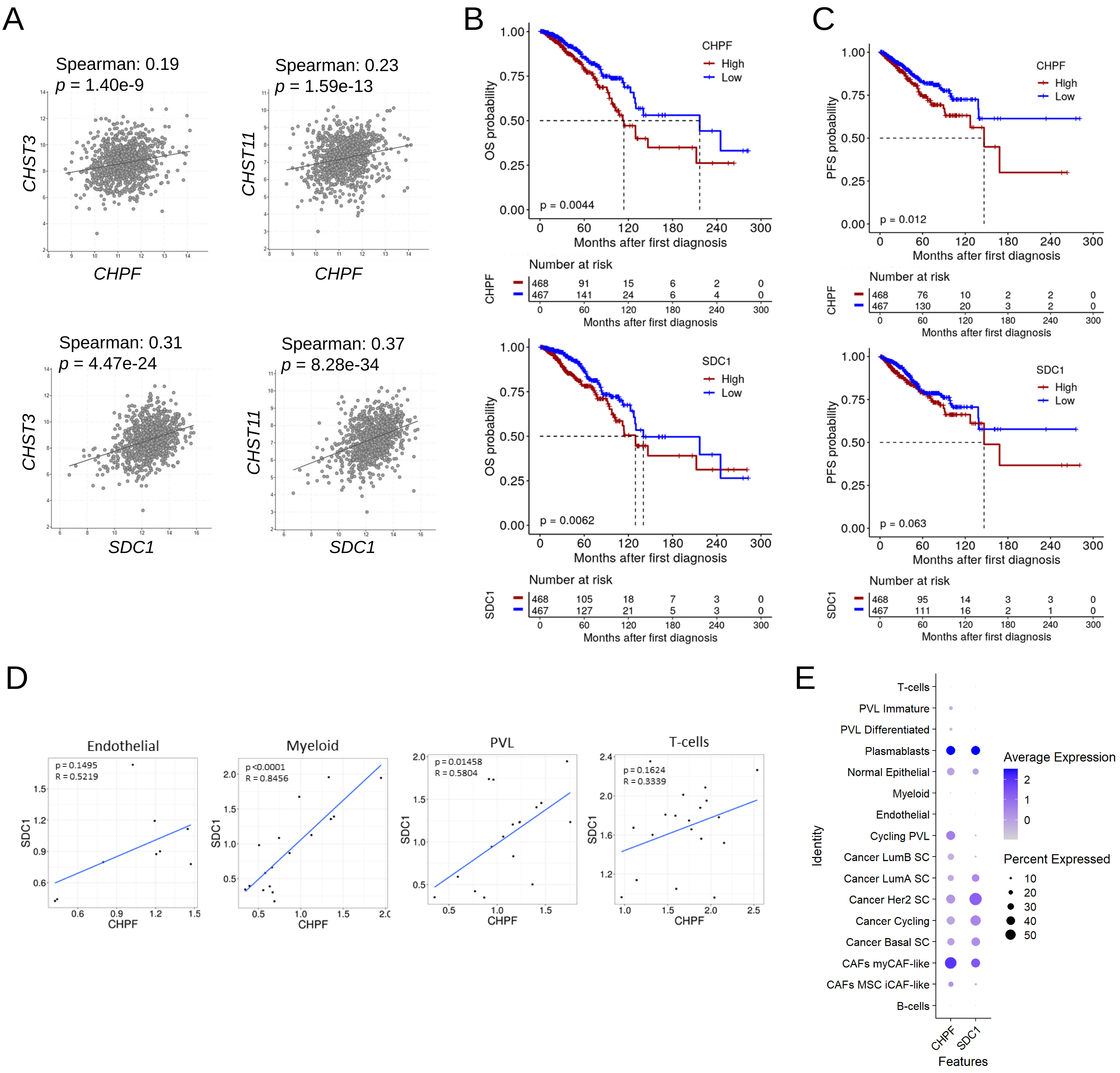

Figure S1. Association of CHPF and SDC1 in breast cancer tissue. (A) Correlation of gene expression of *CHPF* and SDC1 between CS sulfotransferases, *CHST3*, and *CHST11* in human breast cancer tissues. (B) Kaplan-Meier analysis and log-rank test illustrating the overall survival (OS) and (C) progression-free survival (PFS) of breast cancer patients categorized based on their CHPF levels or SDC1 levels. Correlation (D) and expression (E) analysis for CHPF and SDC1 mRNA levels in different types of cells in breast cancer tissue. PVL: perivascular-like cells; CAFs: cancer-associated fibroblasts.

Figure S2

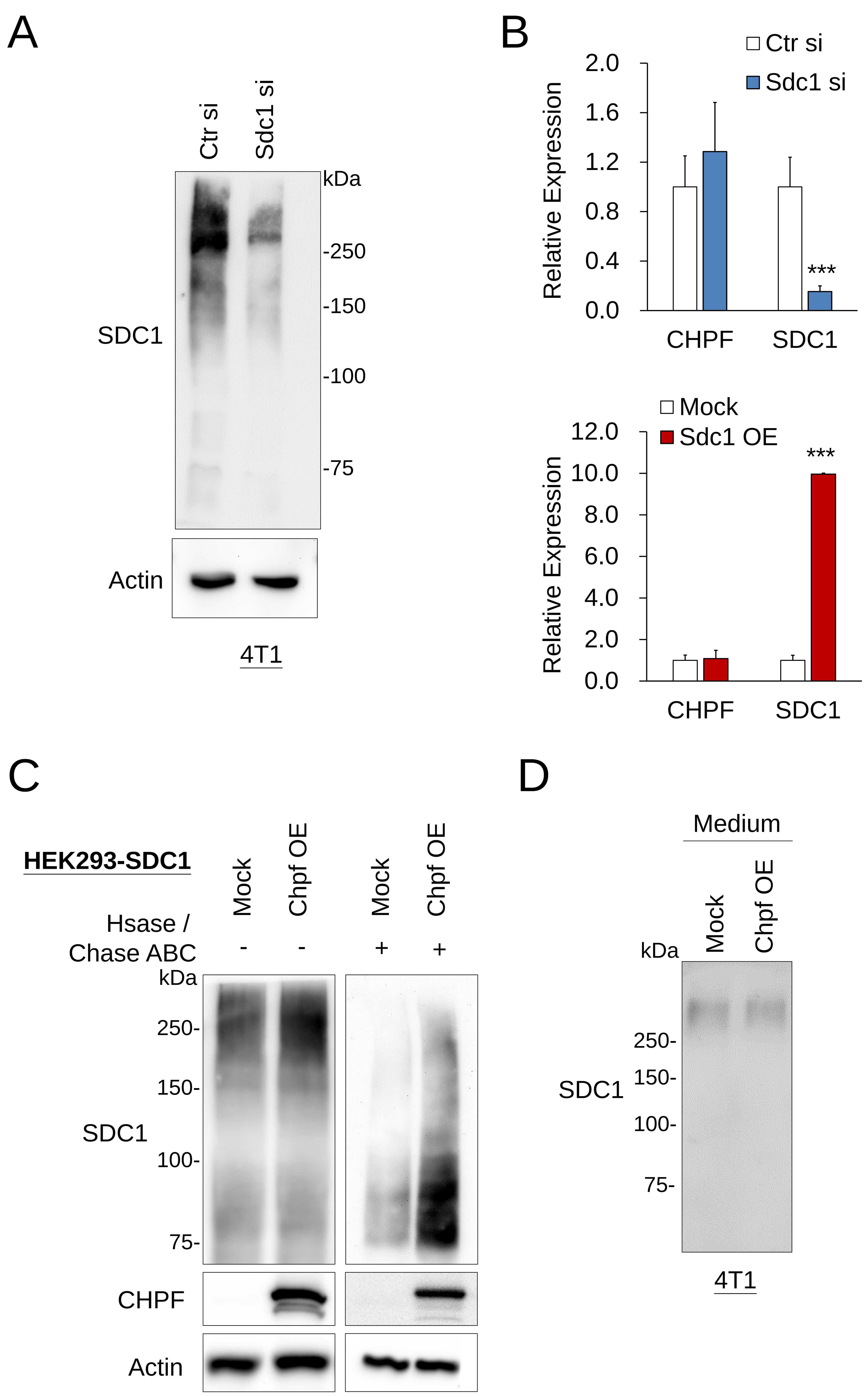

Figure S2. CHPF regulates SDC1 expression. (A) Western blots of control siRNA or Sdc1 siRNA treated 4T1 cell lysate. (B) Silencing of SDC1 or overexpression of SDC1 has no impacts on CHPF gene expression. ***p < 0.001 by two side t-test. (C) Overexpression of CHPF increased SDC1 protein level in HEK293 cells. (D) Western blot of shedding SDC1 in culture medium from mock (empty vector) and Chpf overexpression cells.

Figure S3

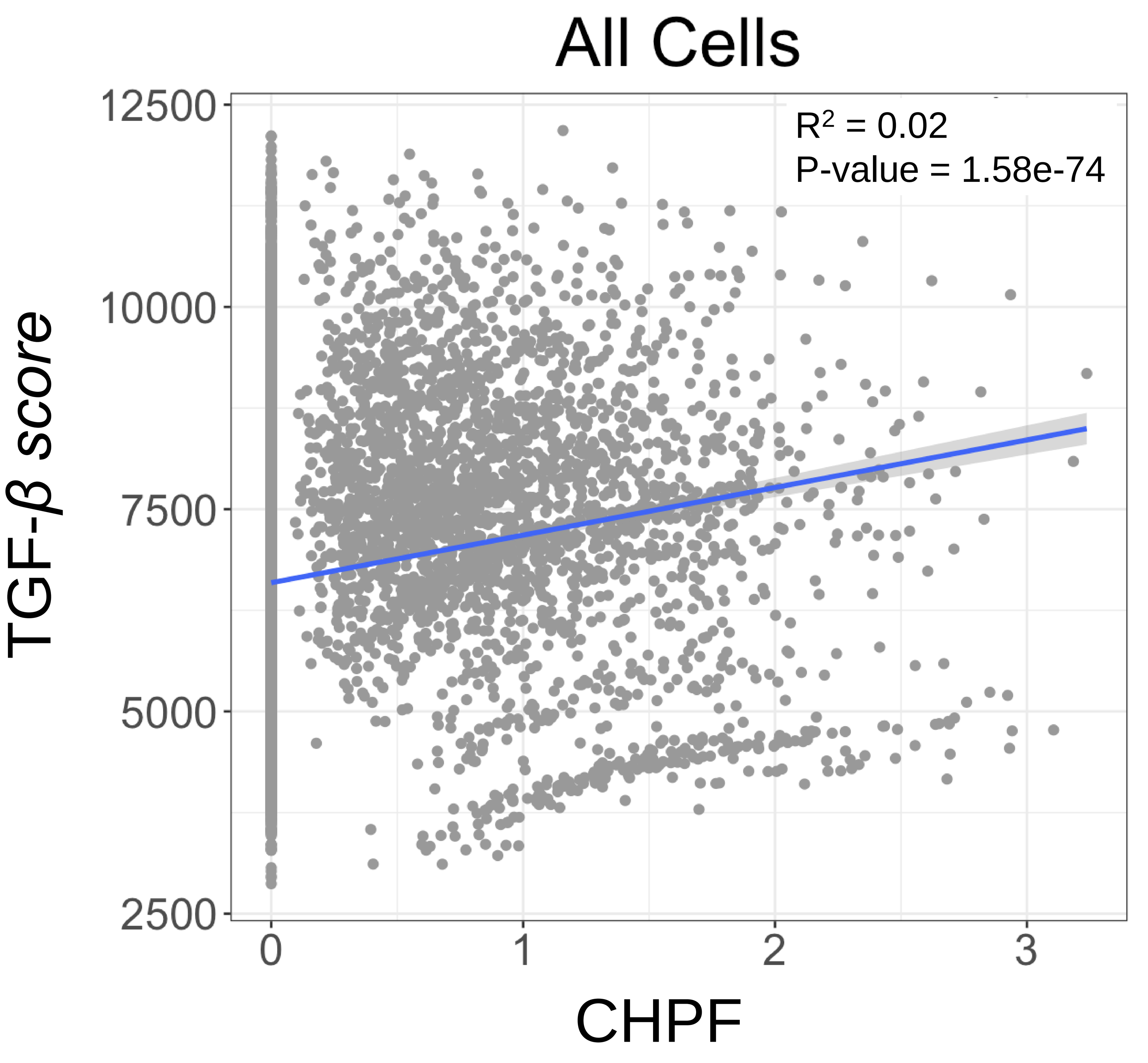

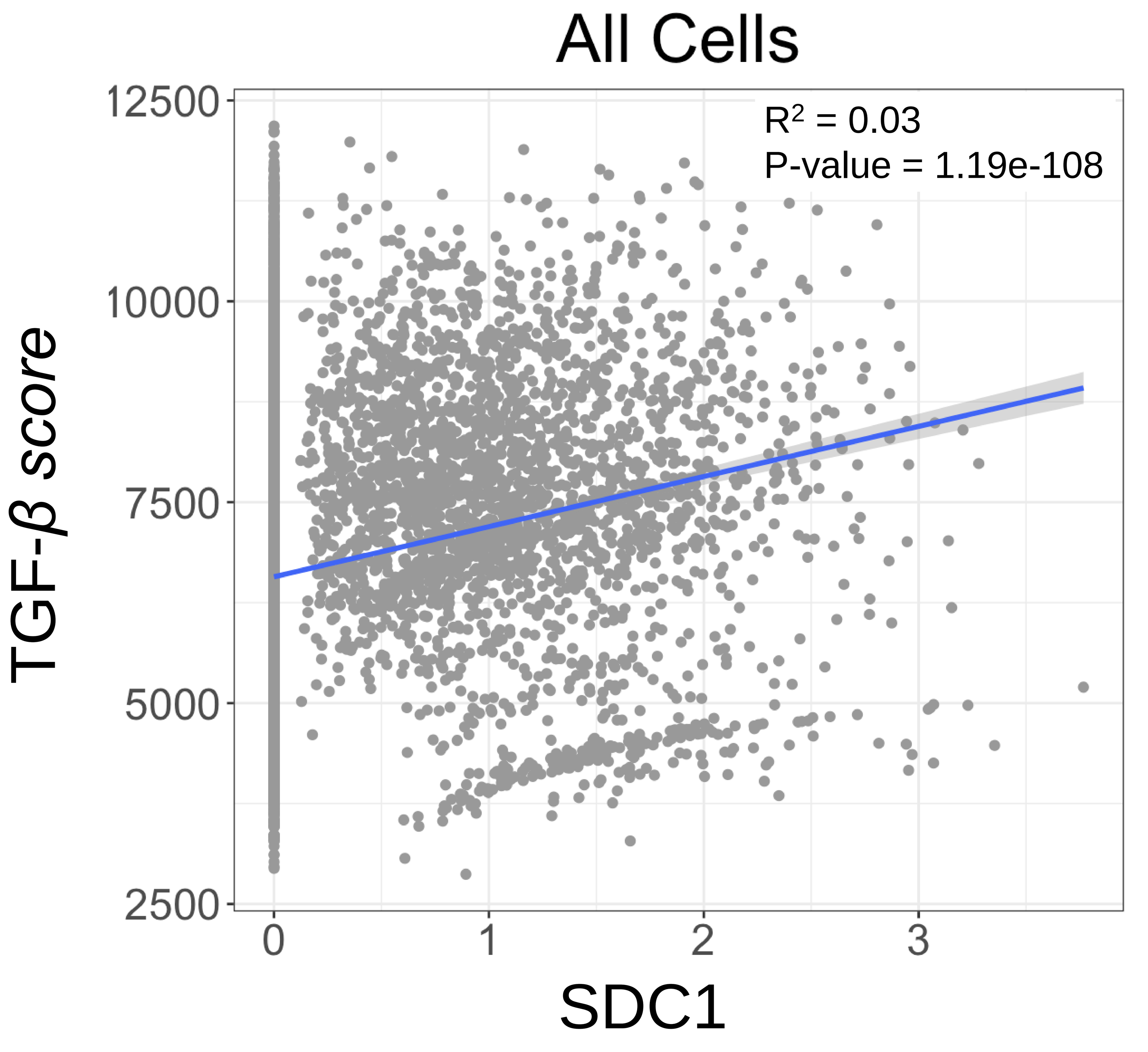

Figure S3. Correlation between TGF-β score and expression of *CHPF* (upper) or *SDC1* (lower).

Figure S4

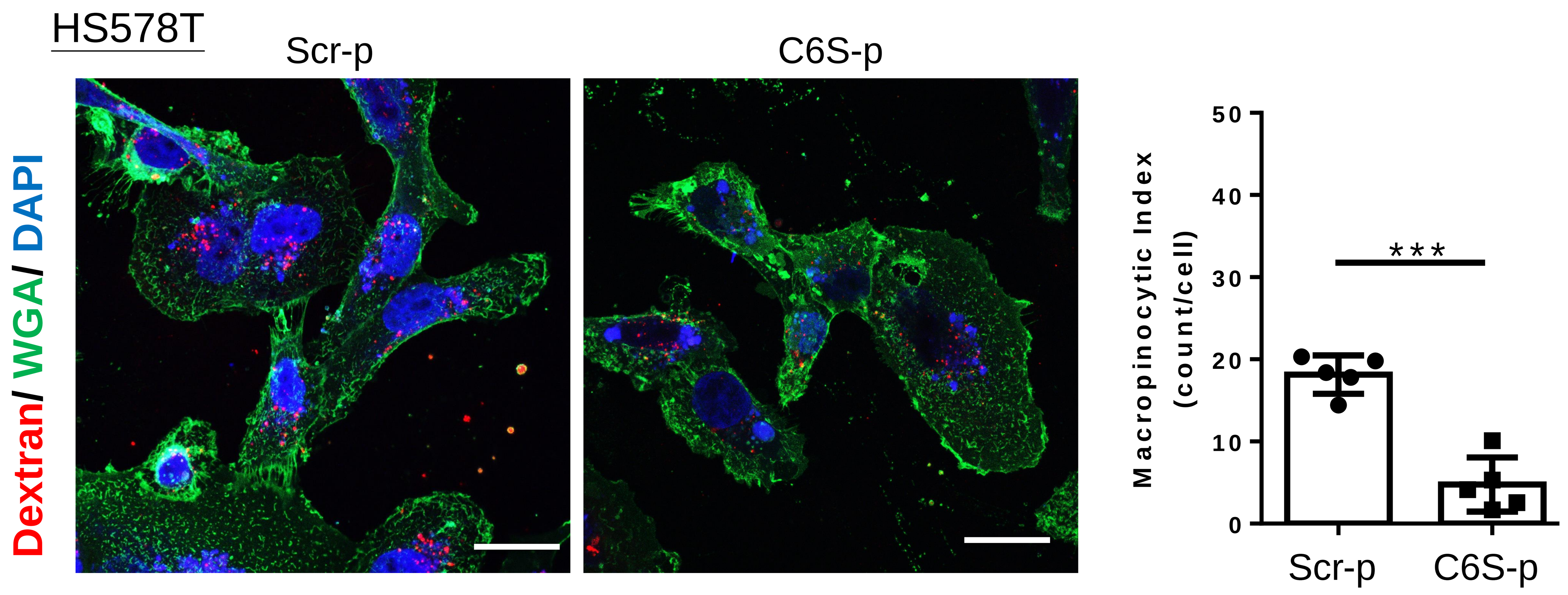

Figure S4. CS-binding peptide suppresses macropinocytosis in HS578T cell. Cells were pretreated with Scr-p or C6S-p for 1 hour, and dextran was added for uptake for 30 min. Wheat germ agglutinin (WGA) was used to label cell membrane (purple). EIPA was used as macropinocytosis suppressor. Mean ± SD was shown from five independent experiments (right). **$p < 0.01$; by two side t-test. Scale bar 20 μm.
